# Pharmaceutical Interventions for Inpatients with Liver Cirrhosis and Liver Transplantation: A Systematic Review of Experimental Studies

**DOI:** 10.3390/jcm12227030

**Published:** 2023-11-10

**Authors:** Nagham Jibai, Alexander Koch, Tom Florian Ulmer, Pia Erdmann, Joachim Andreas Koeck, Albrecht Eisert

**Affiliations:** 1 Hospital Pharmacy, RWTH Aachen University Hospital, 52074 Aachen, Germany; njibai@ukaachen.de (N.J.); pierdmann@ukaachen.de (P.E.); 2 Department of Internal Medicine III, RWTH Aachen University Hospital, 52074 Aachen, Germany; akoch@ukaachen.de; 3 Department of General, Visceral and Transplantation Surgery, RWTH Aachen University Hospital, 52074 Aachen, Germany; fulmer@ukaachen.de; 4 Hospital Pharmacy, Erlangen University Hospital, 91054 Erlangen, Germany; joachim.koeck@uk-erlangen.de; 5 Institute of Clinical Pharmacology, RWTH Aachen University Hospital, 52074 Aachen, Germany

**Keywords:** pharmaceutical care, liver transplantation, liver cirrhosis, experimental study, drug-related problems

## Abstract

Liver cirrhosis, which is considered one of the leading causes of death in the world, can lead to severe complications, and is often followed by a liver transplantation. These patients take an average of nine medications daily. If not managed adequately, it can be accompanied by serious drug-related problems. To reduce this risk, a clinical pharmacist may be included as part of the healthcare team to optimize medication therapy in this population. This study aimed to systematically identify the pharmaceutical interventions which reduced drug-related problems and improved medication therapy for adult hospitalized liver cirrhotic and liver transplant patients when compared to standard care. Three databases (PubMed, Embase, and CENTRAL) were systematically searched from the inception of each database to 25 October 2023, and interventional studies in the English language were included. The risk of bias was assessed according to RoB-I for the UBA study and RoB2 for the identified RCT. The detected interventions to reduce drug-related problems in liver cirrhotic and liver transplant patients were extracted and classified according to a “Hierarchy of Controls” model. Two studies from Germany and the USA met our inclusion criteria, respectively. In these studies, we identified two interventions that included education, expert consultation, and the monitoring of the immunosuppressive medications serum level. The main objective of the two included studies was improving patients’ compliance through adherence. These pharmaceutical interventions identified were classified as administrative controls, which is one of the lowest levels in the “Hierarchy of Controls” with which to address a potential risk. Pharmaceutical interventions to optimize medication therapy were found to be rare in the examined population, and were limited to “administrative controls”. These interventions were limited to transplant patients’ education and the monitoring of the immunosuppressive medication serum levels. No interventional studies were found to have investigated pharmaceutical interventions in patients with liver cirrhosis. Especially regarding this patient group, future studies to reduce DRPs using pharmaceutical interventions are needed. This study received no external funding and its PROSPERO registration number is CRD42022309122.

## 1. Introduction

Liver cirrhosis is an end-stage liver disease and includes many potential complications [1]. Liver transplantation might be considered for adult patients with liver cirrhosis whenever a major complication occurs [2]. The complications of liver cirrhosis include ascites, varices, hepatic encephalopathy, hepatocellular carcinoma, hepatopulmonary syndrome, and coagulation disorders, which can impair patients’ quality of life and decrease their expected years of life [3]. According to the World Health Organization (WHO), liver cirrhosis accounts for 1.8% of all deaths in Europe, causing about 170,000 deaths per year [4]. A total of 75% to 80% of liver cirrhotic cases in Europe are caused by alcohol consumption, which is the third leading risk factor for disease and mortality after tobacco use and high blood pressure [5]. In 2010, liver cirrhosis was ranked among the top 10 leading causes of death in the United States that contributed to years of life lost due to premature mortality [6,7]. The other causes of liver cirrhosis include viral hepatitis, autoimmune diseases, inherited liver diseases, and the inappropriate use of certain medications. According to Weersink et al. [8], patients with cirrhosis take an average of nine medications each day to manage its complications, and around two-thirds of patients use potentially unsafe drugs during their follow-up. This can lead to serious drug-related problems (DRPs). The Pharmaceutical Care Network Europe (PCNE) defined a DRP as “an event or circumstance involving drug therapy that actually or potentially interferes with desired health outcomes” [9]. DRPs constitute a frequent safety issue among hospitalized patients, leading to patient harm and increased healthcare costs [10]. DRPs include the choice of drug, drug dosage, adverse drug reactions, drug interactions, the lack of monitoring of a drug’s effects, toxicity, and adherence problems, which can be actual or potential [11]. The pharmacokinetic and pharmacodynamics characteristics are altered in patients with cirrhosis due to the functional abnormalities of the hepatocytes [12]. Furthermore, DRPs occur frequently in liver cirrhotic patients; Franz et al. found in a Swiss retrospective study that over one-fifth of patients were affected by one or more potential drug–drug interactions (pDDI, 86/400 patients) [13]. Of 132 pDDIs, 3 resulted in a hospital admission. In another study which used the same patient population, nearly half of all the patients received medication doses which were not appropriate (184/400 patients) [14]. In a 10-bed medical intensive care unit at a tertiary care hospital in India, the medication charts for 78 patients with decompensated liver cirrhosis were retrospectively reviewed. This identified an incidence rate of DRPs of 298 per 1000 patients [15]. The most common type of DRPs were pDDIs, but almost ten percent of the DRPs contained a contraindicated medication. It is not only liver cirrhotic, but also liver transplant patients, who are affected by high frequencies of DRPs. This was illustrated by a cohort study in Saudi Arabia, which included 255 liver transplant patients [16]. The patients were affected by 407 medication errors (1.6 errors/patient). Most of those errors were unrelated to the immunosuppressants (87%), indicating the need to include all prescribed medications in a pharmaceutical care program, not only those in the immunosuppressants regimen.

To reduce the risks of DRPs, a clinical pharmacist may be involved regularly as a part of the healthcare team in different settings, contributing to the optimization of drug therapy and the prevention of DRPs [11,17]. Recently, Mulder et al. compared two cohorts of Dutch liver transplant patients for DRPs, one with and one without ambulatory pharmaceutical consultation (94 and 197 patients, respectively) [18]. The cohort with pharmaceutical consultation showed significantly less use of suboptimal or contraindicated therapies (2.4% vs. 9.5% of DRPs, e.g., use of naproxen for headache) and unnecessary drugs (17.3% vs. 58.7% of DRPs, e.g., the use of three low-dosed antihypertensive drugs for well-regulated blood pressure).

**Figure 1:**
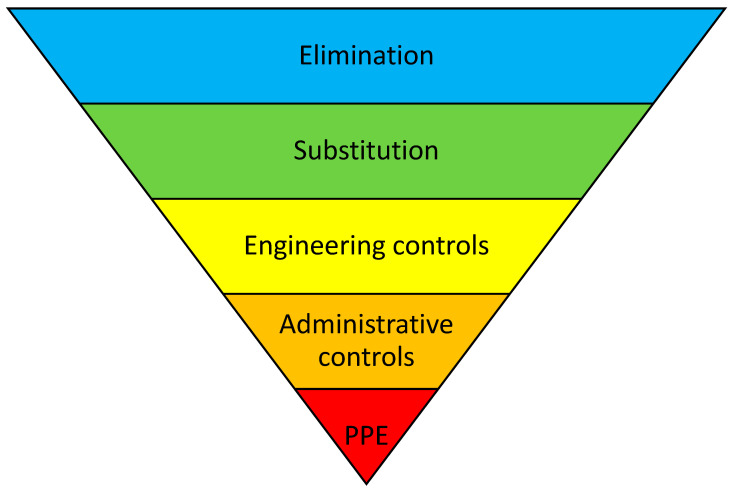
“Hierarchy of Controls”, including personal protective equipment (PPE). Adapted from the National Institute for Occupational Safety and Health (NIOSH), Washington, DC, USA.

This review not only performed a search to summarize the existing evidence for pharmaceutical interventions in liver transplant patients, but it also sought to classify the intervention type. To do so, the “Hierarchy of Controls” was used, which is a five-step approach to control risks (e.g., with medication therapy) [19]. It ranks risk control methods from the highest level of protection and reliability to the lowest and least reliable protection as follows: “Elimination-“, “Substitution-“, “Engineering-“, and “Administrative Controls”, followed by “Personal Protective Equipment”, as shown in Figure 1.

This systematic review identifies and evaluates the pharmaceutical interventions that improved medication therapy in adult liver cirrhotic and liver transplant patients, by reducing DRPs when compared to standard care.

## 2. Materials and Methods

For the reporting of this systematic review, the PRISMA 2020 guidelines were followed [20]. The review was registered with PROSPERO (reg. no. CRD42022309122) and with the ethics committee of the University RWTH Aachen (EK 23-048).

### 2.1. Eligibility Criteria and Definitions

This systematic review included all studies published in the English language that investigated the pharmaceutical interventions in an intervention group (pharmaceutical intervention) compared to a separate control group (standard care).

All the human interventional studies that investigated pharmaceutical interventions to reduce DRPs in the treatment of adult liver cirrhotic patients (≥18 years old) and/or liver transplant patients in a hospital setting were considered. Only the studies that included liver transplant patients (no other types of transplantations) and/or liver cirrhotic patients were included. Reviews, case reports, journal letters, journal notes, commentaries, and editorials were excluded, as were studies that only presented descriptive results without separate control and intervention groups, and studies that only addressed the economic impacts. The definitions of the study types were adopted from the Cochrane Effective Practice and Organization of Care Review Group [21]. A randomized controlled trial (RCT) was defined as an experimental study in which the people involved receive random types of interventions. An uncontrolled before–after study (UBAs) was defined as a study that involves an interventional group and a control group.

A “Pharmaceutical intervention” was defined as a recommendation initiated by a pharmacist in response to a DRP occurring in an individual patient in any phase of the medication-use process [17]. The Pharmaceutical Society of Australia has defined a clinical intervention as “any professional activity by the pharmacist directed towards improving the quality use of medicines and resulting in a recommendation for a change in the patient’s medication therapy, means of administration or medication-taking behaviour” [22]. These interventions were classified according to the “Hierarchy of Controls”, a method suggested by the United States National Institute for Occupational Safety, to help to arrange interventions in a sequential order through different levels [19]. It ranks risk control methods, from the highest level of protection and reliability to the lowest and least reliable protection, as follows: “Elimination-”, “Substitution-”, “Engineering-”, and “Administrative Controls”, followed by “Personal Protective Equipment”, as shown in Figure 1. The first three steps may be summarized as the “higher levels” of control that have shown a 1.5 higher chance of resulting in significantly reduced error rates compared to the remaining two steps, which are summarized as the “lower levels” of control [19]. Originating from within occupational safety, the method was adapted to healthcare risk management by Card et al., and subsequently to medication safety by Koeck et al. [23,24].

### 2.2. Information Sources

Three databases were searched (Medline, Embase, and CENTRAL) for studies that investigated pharmaceutical interventions in liver transplant patients and liver cirrhotic patients in a hospital setting. Manual reference checks were carried out to look for missing studies, but none met our inclusion criteria.

### 2.3. Search Strategy

The research spanned the interval from each database’s inception to 25 October 2023. The terms for the searches in Medline, CENTRAL, and Embase were compiled and pilot-tested before the searches, and included subject headings and keywords. For each database, an adapted version of the search term was used (Supplementary Table S1). No automatic filters were activated for the search terms; only medical subject headings and keywords were used.

### 2.4. Selection Process

Two reviewers (N.J. and J.A.K.) independently screened all the references (titles and abstracts) that were retrieved using the search strategy. Two reviewers (N.J. and A.E.) rescreened new articles until 25 October 2023, but no relevant articles were found. To do this, a previously published abstract screening form was pilot-tested that contained nine exclusion criteria (Supplementary Table S2) [25]. When no exclusion criterion was applied, the abstract was considered for the data collection process. The results of the abstract screening were analyzed using Excel 2016 (Redmond, WA, USA). The interrater agreement was calculated via Cohen’s Ƙ [26].

### 2.5. Data Collection Process

For all the abstracts that did not fulfill the exclusion criteria, the full texts were retrieved. Initially, these full texts were checked against the above-mentioned exclusion criteria. When the full texts seemed eligible, a detailed collection of the data items followed. The first reviewer (N.J.) performed the data collection, and the second reviewer (J.A.K.) amended these results when necessary. In the case of disagreement, this was resolved by discussion. A third party (A.E.) was contacted when no consensus was reached. Each full text was characterized as having either a single intervention or a bundle of interventions. The results were analyzed using Excel 2016 (Redmond, WA, USA). 

### 2.6. Data Items

The extraction of the data from the enclosed articles included the publication details, the country of the study’s performance, the characteristics of the included patients, the study design, and the intervention(s)’ number, type, and effect.

### 2.7. Study Risk of Bias Assessment

The risk of bias in the studies included was independently assessed by two reviewers to reduce bias and the potential for errors. N.J. and P.E. assessed ROBIN-I; RoB2 was determined by N.J. and J.A.K. using the Cochrane risk of bias tool framework. The ROBINS-I tool was used to rate the risk of bias in the non-randomized studies, and the RoB2 tool to evaluate the randomized studies [27].

### 2.8. Effect Measures

The effectiveness of the treatments was determined by separately calculating the error rate for the control and intervention groups, where applicable. Based on this, the “Absolute Risk Reduction” (ARR) for the main outcome of each study was calculated (Table 1). The outcomes are described in Section 3.4. 

### 2.9. Synthesis Methods

Each intervention was independently classified by two of the authors (N.J. and J.A.K.), according to the “Hierarchy of Controls” model [19]. In the case of varying results, discrepancies were resolved by a discussion. A third party (A.E.) was contacted when no consensus was reached. The “Hierarchy of Controls” model classifies interventions into five categories, according to the assumed potential for risk reduction (Figure 1). “Elimination” is considered the most effective type of intervention with which to reduce risk. “Substitution”, the second category in the hierarchy, is the substitution of hazardous methods or materials for less hazardous ones, thus reducing the risk of DRP [30]. One example of this is implementing a computerized physician order entry to reduce medication errors, in comparison to paper-based prescribing [31]. “Engineering controls” involve isolating hazards or altering how tasks are carried out to lower the risk of hazards, e.g., a Clinical Decision Support System that warns a provider before processing an overdosed medication [25]. “Administrative controls”, the fourth step, have a lower protection level from hazards as compared to the others. They do not eliminate risks; rather, they minimizes the exposure to hazards, e.g., through educational supply and training procedures. The fifth and lowest level is “personal protective equipment”.

## 3. Results

### 3.1. Study Selection

**Figure 2:**
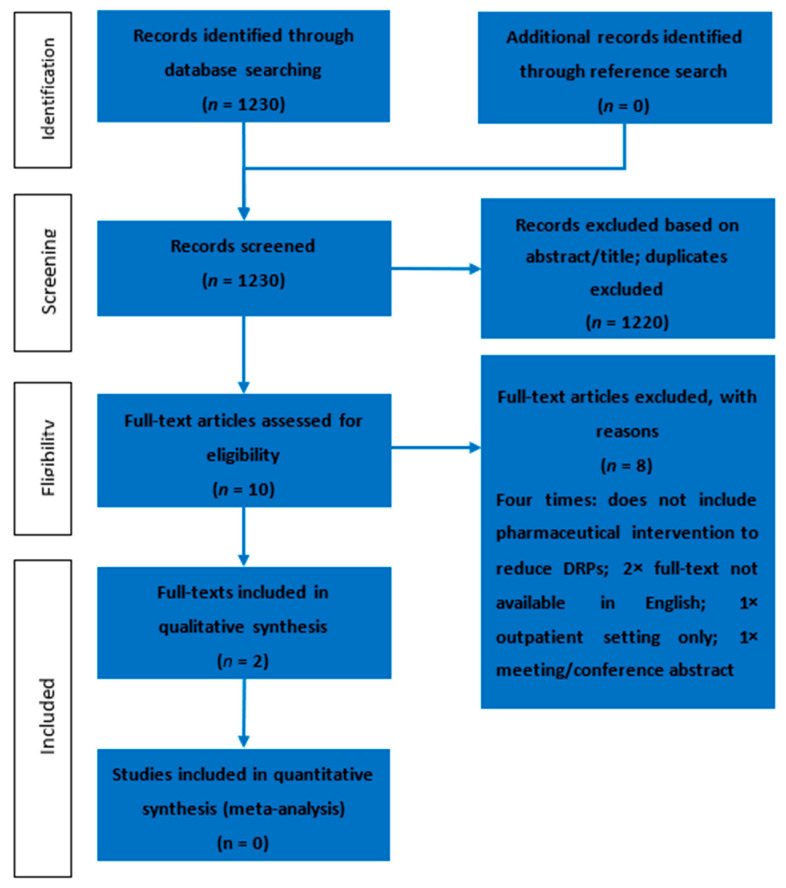
Results from the search and selection process, displayed using a PRISMA flow diagram.

After screening 1230 abstracts, 10 full texts were eligible for further full-text screening, according to the exclusion criteria (Figure 2). Of these 10 full texts, only two described pharmaceutical interventions with which to reduce DRPs in an experimental context and, thus, were included in this review (Table 1). The interrater agreement between the two researchers to include or exclude an abstract was calculated via the weighted Cohen’s κ. This resulted in a coefficient of 0.57, which is a “moderate” agreement, according to the nomenclature.

### 3.2. Study Characteristics

The two full texts included in this review represent two studies originating from Germany and the United States [28,29]. One RCT was identified and one UBA. Both studies investigated liver transplant patients, but no studies were identified that addressed patients with liver cirrhosis in an inpatient setting. The studies were single-center studies in a university hospital and a tertiary care hospital (Table 1). Both studies comprised inpatients per the inclusion criteria; one of them also included follow-up care [28,29]. The studies durations comprised the first three and twelve months after liver transplantation, respectively [28,29].

### 3.3. Risk of Bias in Studies

**Figure 3:**
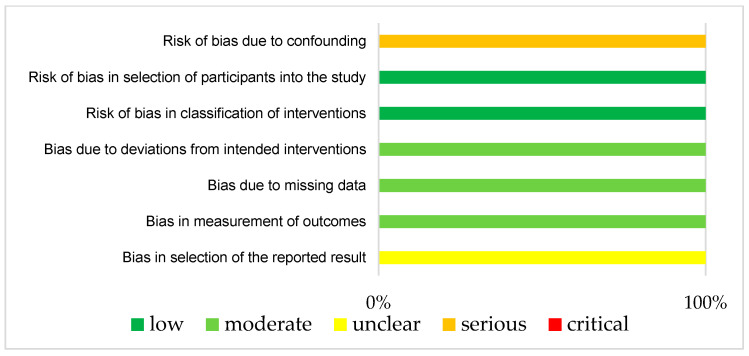
Bias risk assessment (Robins-I) for the included UBA (Schuh et al. [29]).

**Figure 4:**
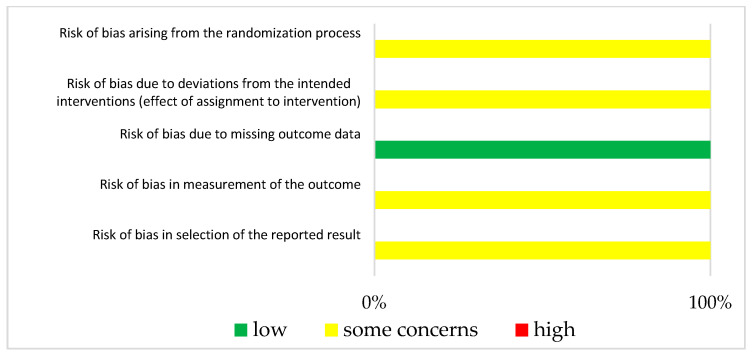
Bias risk assessment (RoB2) for the included RCT (Klein et al. [28]).

The results of ROBINS-1 and RoB2 are represented in Figure 3 and Figure 4, respectively. Both studies had small study sizes. The RCT (Klein et al. [28]) did not report the randomization process. Neither the sequence generation nor the allocation concealment were clarified, which resulted in some concerns about bias [27]. As randomization is not applicable for UBAs, potential confounding has to be elaborated upon in the study design. Unfortunately, this was not the case in the “Clinical Note” provided by Schuh et al. [29]. This introduced a potentially serious risk for bias. For example, we could not identify whether one or more clinical pharmacist/s performed the consultation visits (introducing the need for a consistent level of patient training). Additionally, other potential determinants of a differing tacrolimus level were not mentioned in this publication.

### 3.4. Results of Individual Studies

One study investigated the patients’ compliance with immunosuppressive therapy as the primary outcome [28]. Compliance was defined through the number of correct “medication event monitoring system” (MEMS) bottle openings per all monitored days. To investigate this, patients who underwent liver transplantation between September 2003 and January 2005 in one German transplantation center were randomly assigned to routine care or an intervention group; the intervention group contained a pharmaceutical care program. The pharmaceutical care program started one week before discharge from the hospital, and included an educational approach. One clinical pharmacist met the patient three to four times during the last week of their stay, and mentioned topics like immunosuppressive drug effects/side effects, interactions, vital signs, and laboratory data. On the day of discharge, the clinical pharmacist handed out the discharge medication plan, as well as information regarding immunosuppressive therapy and a diary for documenting vital signs and laboratory data. In the first year after transplantation, the clinical pharmacist met the patient at least every three months to discuss medication or laboratory changes, as well as drug-related problems. Twenty-four and twenty-six patients were assigned to the two groups, of which twenty-one patients were evaluated for the primary outcome. Ninety percent of those in the interventional group (18/20 patients) showed compliance with the immunosuppressive therapy, compared to 57% of the control group (12/21 patients). This corresponds to a 33% absolute risk reduction for the intervention group compared to the control group. The secondary outcomes underlined the primary outcome, e.g., by an increased number of immunosuppressant serum drug concentrations in range. Of the 121 tacrolimus serum concentrations in the control group, 51% (*n* = 62) were assessed to be in the therapeutic range, compared to 78% in the intervention group (98/125 serum concentrations). This corresponds to an absolute risk reduction of 27%.

The second study Investigated the therapeutic drug levels of tacrolimus before and after the pharmaceutical interventions [29]. Seventy-four patients were included who received a liver transplantation at one center in the United States during the period from March 2015 to March 2016. The first twenty-one days after transplantation were named as the control period, and days 22–120 after transplantation as the intervention period. Three to seven days after transplantation, a pharmacist consulted the patient to educate them on tacrolimus medication regimens and on the interacting drugs, foods, and herbals. The pharmacist addressed drug adherence via the assessment of medication fills/refills, and provided education about pillboxes, the time intervals between drugs and food, and reminder alarms for medication administration. In the first 120 days after transplantation, the patient remained near the transplantation clinic to ensure proper immunosuppressive drug titration. Seventy-four patients were included. Before the pharmacist post-transplant consultation, 25% of the tacrolimus drug levels were in the therapeutic range. After consultation, this percentage rose to 49%. Thus, the intervention led to an absolute risk reduction of 24%.

### 3.5. Synthesis of Results

Two different pharmaceutical interventions were identified to reduce DRPs. Both studies represented single intervention studies and focused on interventions that had educational purposes [28,29]. They led to comparable results, with absolute risk reductions of 24 and 33%. In addition, the “tacrolimus levels in range” were considered a secondary outcome in the study of Klein et al. [28], and a primary outcome in the study of Schuh et al. [29]. Both studies resulted in comparable absolute risk reductions with respect to this item (27% and 24%).

The pharmacist interventions included patient education about their individual medications pre- and post-transplantation, and lifestyle-related education [29], in addition to the monitoring of patients’ compliance and adherence through their follow-up care [28,29]. In one study, the clinical pharmacist optimized the medical therapy through participation in the medical rounds with the medical team, and was responsible for screening the CYP3A4 interacting foods, medications, and herbal supplements in a hospital setting [29]. For the measurability of the intervention, serum concentration controls were used in both studies.

Both studies implemented educational interventions that are categorized as “administrative controls” according to the “Hierarchy of Controls”, and the “lower levels” of control according to Koeck et al. [19,23].

## 4. Discussion

Our systematic review is the first to use the “Hierarchy of Controls” to investigate pharmaceutical interventions to reduce drug-related problems in liver cirrhotic and liver transplant patients. At present, liver transplantation remains the only curative option for a selected group of patients, but pharmacological therapies are currently being developed that could delay or stop the progression to decompensated cirrhosis, or even reverse cirrhosis [32]. The mortality from liver cirrhosis in 187 countries worldwide increased from around 676,000 in 1980 to over 1 million in 2010 [33]. Weersink et al. stated that 60% of the drugs used in liver cirrhotic patients were potentially unsafe after their classification using a previously published practical guidance [8]. This guidance classified every drug according to its potential safety risks (i.e., safe, no additional risks known, additional risks known, unsafe, and unknown) and, if applicable, specific dosing advice was also given [8], indicating the potential impact of a pharmacist contribution. Drugs were classified as safe when no increase in harm was found compared to persons without liver cirrhosis [34]. Unfortunately, no experimental studies were identified that assessed the benefit of a clinical pharmacist for liver cirrhotic patients concerning the reduction in DRPs.

Furthermore, although this topic is very relevant, we could only identify two experimental studies in transplant patients [28,29]. This result is underlined by a systematic review that evaluated pharmaceutical interventions in solid-organ transplantations [35]. This publication included only one article focused on liver transplantation, which is also included in our review [28]. 

The two studies included here were limited to educational interventions and healthcare team counseling, which led to fewer rejection episodes [28] and showed an increased achievement of target serum concentrations in immunosuppressive therapy [31]. Although the mentioned interventions were limited to pharmaceutical consultations, they succeeded in reducing DRPs by enhancing patients’ adherence. A retrospective cohort study showed that 22–62% of liver transplant patients reported non-adherence [36]. Another retrospective study concluded that 1 in 10 transplant patients might have died from poor drug adherence after liver transplantation [37]. Pharmacists play a major role in a multidisciplinary team, by enhancing the patients’ and caregivers’ understanding of the importance of immunosuppressive therapy [38].

All of the interventions mentioned in our two selected studies are classified as “administrative controls”, which is one of the lowest levels for reducing the risk of error in the hierarchy. Card et al. discussed that interventions may be best performed at the three highest levels of controls (i.e., elimination, substitution, and engineering controls) [24]. Examples of “higher level” interventions are, e.g., patient ID reentry at drug prescribing, the implementation of a computerized prescribing order entry, or a Clinical Decision Support System with alert functions [23]. According to Manuele et al., the actions described as being in the “higher levels” of control are more effective, because they: (1) are preventive actions that reduce risk by using design and substitution measures; (2) rely the least on personnel performance; and (3) are less defeatable. The actions described as being in the “lower levels” of control rely greatly on the performance of people [30]. Furthermore, the use of interventions from the “higher levels” of control were associated with a 1.5- and 1.6-times higher chance of resulting in significantly positive results, respectively [23,24]. However, in a recently published systematic review that included pharmacists’ interventions with diverse types of transplant patients, all the described interventions were in the “lower levels” of control (e.g., pharmaceutical care service, interviews, and patient education) [35]. Thus, intervention types should be developed and tested that support patient safety via “higher level” interventions, e.g., a mandatory medication approval (engineering control) or the substitution of tacrolimus dosage forms that have to be administered two times daily by those that have to be administered once daily only (substitution control). These “higher level” interventions may be accompanied by educational campaigns, because a recent review concluded that bundles of interventions might be more effective than single interventions [23]. Despite this, experienced clinical pharmacists in the field should train those who would build up a pharmaceutical care program in liver transplant centers. The British Association for the Study of the Liver founded a specialist pharmacy group, the “British Hepatology Pharmacy Group”, to optimize the medication of patients with liver diseases via supporting colleagues [39]. Furthermore, the American Society of Health-System Pharmacists published a report on transplant pharmacy services, to give a guideline for the minimal and optimal services that should be provided by local transplant pharmacists [40]. In this guideline, it is pointed out that all transplant pharmacy services should be rendered compliant with national and/or local requirements. However, the transplant pharmacy service may be divided into three phases: the first is the pre-transplantation phase, the second is the peri-operative phase, and the third is post-transplantation and the ambulatory management setting. As of now, clinical pharmacists in the United States do not have a standard of practice for the provision of transplant pharmacy services, indicating a worldwide need for the building up and structuring of such pharmacy services. Schuh et al. mentioned this three-part service in their study [29]. In this specific context, pharmacist participation pre-transplant was required, so post-transplantation involvement was built up by those colleagues. In other circumstances, like in the European system, no requirements for transplant pharmacists exist as of now. Only a few transplant pharmacists are in place, e.g., in German transplant centers; the implementation of services and the networking of German and European solid-organ transplant pharmacists is currently needed. When focusing on European bone and marrow transplantation centers, 75 centers reported having a clinical pharmacist or pharmacologist as part of the interdisciplinary team [41]. The European Society for Blood and Marrow Transplantation lists 647 centers for 2022. Thus, a minimum of 12% of all centers are equipped with a dedicated pharmacist. For solid-organ transplant pharmacists, the corresponding data are not known.

The primary outcomes were identified as surrogates for patient clinical outcomes; namely, the percentage of days with the correct bottle openings of immunosuppressive drugs [28], as well as tacrolimus drug levels in range [28,29]. Direct patient-centered outcomes were not identified in the two studies; those outcomes were described in a systematic review to be “patient satisfaction”, “patient clinical outcomes”, and “organizational outcomes” [42].

The strength of our review is that we use, for the first time, the “Hierarchy of Controls” to classify the pharmaceutical interventions with which to reduce drug-related problems in liver cirrhotic and/or liver transplant patients. Using this tool, we can elaborate on the level of pharmaceutical interventions in this patient group for the first time.

In addition, there are limitations to mention. We included only studies that solely focused on liver cirrhotic and/or liver transplant patients. Studies with liver and other solid-organ transplantations were excluded. Although patients with liver transplantation receive similar immunosuppressive therapy compared to, e.g., kidney transplantation, there are some differences. The main difference is that there are far more kidney transplantations compared to liver transplantations. In 2022, there were more than two times more kidney transplantations registered in eight European countries as compared to liver transplantations (4025 vs. 1500 living and deceased donor transplantations, respectively [43]). In addition, the number of kidney transplantation centers in eight European countries is double that of liver transplantation centers (69 vs. 38 centers, respectively) [44]. These numbers also reflect the scientific output; in a current systematic review about the impact of transplant pharmacists, three-fourths of the included studies addressed kidney transplant patients (8/12 studies). Only one study addressed liver transplant patients. Thus, enlarging the inclusion criteria to include studies that were not solely focused on liver transplant patients would have based our results mainly on other types of transplantations.

However, this focus on studies of solely liver cirrhotic and/or liver transplant patients led to two studies only. Our results should, therefore, be interpreted cautiously.

## 5. Conclusions

This is the first study that has investigated pharmaceutical interventions in liver cirrhotic and liver transplant patients using the “Hierarchy of Controls”. The identified interventions were limited to “administrative controls”, one of the lowest levels, and included education and monitoring of the immunosuppressive medications. Studies that focused on pharmaceutical care in liver transplant patients are rare, and investigations of liver cirrhotic patients are altogether missing. In addition, investigations into pharmaceutical care for drug classes other than immunosuppressant drugs are needed.

## Figures and Tables

**Table 1:** Summary of study characteristics. CG = control group; IG = intervention group; MEMS = medication event monitoring system.

First Author (Year of Publication)	Country	Study Type	Study and Patient Setting	Population and Age Group	Gender	Standard Care	Number of Intervention(s), Description of Intervention(s)	“Hierarchy of Controls”	Results	Effect of Primary Outcome [%]
Klein, A. (2009) [28]	Germany	RCT	University hospital, inpatient, and follow-up care.	Liver transplant patients. CG—24 patients; IG—26 patients. Age: CG mean, 50.1 years; IG mean, 52.8 years.	CG: Female: 11 (46%) Male: 13 (54%) IG: Female: 12 (46%) Male: 14 (54%)	Routine clinical care.	Single intervention. Pharmaceutical care program: − One week before discharge, meet with patient 3–4 times. Education about drug action, side effects, interactions, vital signs, laboratory data, and discharge medication. − On discharge: discharge medication plan, information regarding immunosuppressive therapy, diary for vital signs, and laboratory data. − During the first year after transplantation: meet with the patient at least once per quarter, at most monthly. Discussion about medication changes, laboratory values, and DRPs.	Administrative Controls	Primary outcome: patients’ compliance with immunosuppressive therapy, defined as the number of correct MEMS bottle openings per all monitored days; ≥80% was seen as compliant. CG: data from 21 patients were available and 12 patients were compliant (57%). IG: data from 20 patients were available and 18 patients were compliant (90%). Secondary outcomes: − Pill counts: Data from 24 patients in each group were available. The CG had 14/24 patients and the IG had 24/24 patients, with a mean compliance rate ≥90%. − Immunosuppressant drug concentrations: Data from 24 patients in each group were available. The CG had 62/121 (51%) and the IG had 98/125 (78%) of the serum concentrations in the target range. − Morisky score (MMAS-4): there are 4 questions; a “no” answer means compliance. CG: after a study duration of 12 months, 12/19 patients answered “no” for all 4 questions (63%). IG: 20/23 patients (87%). − Self-report: the number of forgotten doses in the last 4 weeks. CG: after a study duration of 12 months, 12/19 patients declared no forgotten doses (63%). IG: 19/23 patients (83%).	ARR = 33%
Schuh, MJ (2018) [29]	USA	UBA	Tertiary care and inpatients.	Liver transplant patients. 74 patients. Age: mean, 59.7 years	Female: 29 Male: 45	Routine clinical care with pre-transplant pharmacist consultation.	Single intervention. Post-transplant face-to-face pharmacist consultation (medication adherence monitoring; screening for CYP3A interacting foods, medications, and supplements; and education of patients/caregivers).	Administrative Controls	Primary outcome: the percentage of tacrolimus drug levels in range. CG (tacrolimus drug levels in range three weeks before the post-transplant pharmacist consultation): 25% of drug levels in range (5–10 ng/mL). IG (tacrolimus drug levels in range four months after the post-transplant pharmacist consultation): 49% of drug levels in range.	ARR = 24%

## Data Availability

Data are available upon request to the corresponding author.

## Supplementary Materials

The following supporting information can be downloaded at: https://www.mdpi.com/article/10.3390/jcm12227030/s1, Supplement Table S1: Search terms that were used for this systematic review, adapted to each database. Supplement Table S2: Nine exclusion criteria for abstract screening.
