# Supplementary material for: Pharmaceutical Interventions for Inpatients with Liver Cirrhosis and Liver Transplantation: A Systematic Review of Experimental Studies
Source: J Clin Med. 2023 Nov 10;12(22):7030. doi: 10.3390/jcm12227030 (PMC10671912; doi:10.3390/jcm12227030)
Supplement: Supplementary file 1 [file jcm-12-07030-s001.zip › jcm-2665557-supplementary.pdf]

Supplement

Pharmaceutical Interventions for Inpatients with Liver Cirrhosis and Liver Transplantation: A Systematic Review of Experimental Studies

Nagham Jibai [1], Alexander Koch [2], Tom Florian Ulmer [3], Pia Erdmann [1], Joachim Andreas Koeck [4] and Albrecht Eisert [1,5,*]

1 Hospital Pharmacy, RWTH Aachen University Hospital, 52074 Aachen, Germany; njibai@ukaachen.de (N.J.); pierdmann@ukaachen.de (P.E.)
2 Department of Internal Medicine III, RWTH Aachen University Hospital, 52074 Aachen, Germany; akoch@ukaachen.de
3 Department of General, Visceral and Transplantation Surgery, RWTH Aachen University Hospital,
52074 Aachen, Germany; fulmer@ukaachen.de
4 Hospital Pharmacy, Erlangen University Hospital, 91054 Erlangen, Germany; joachim.koeck@uk-erlangen.de
5 Institute of Clinical Pharmacology, RWTH Aachen University Hospital, 52074 Aachen, Germany
* Correspondence: aeisert@ukaachen.de

Database	Search term
Medline	"Liver cirrhosis"[MeSH Terms] OR "Liver Transplantation"[MeSH Terms] OR ("liver*"[Title/Abstract] AND "cirrhos*"[Title/Abstract]) OR ("liver"[Title/Abstract] AND "transplant*"[Title/Abstract]) AND ("pharmacists"[MeSH Terms] OR "pharmaceutical services"[MeSH Terms] OR "pharmacist*"[Title/Abstract] OR "transplant pharmacist"[Title/Abstract] OR "Medicines Management"[Title/Abstract] OR "Medicines Optimisation"[Title/Abstract] OR "Medicines Optimization"[Title/Abstract])
Embase	('liver transplantation'/exp OR 'liver'/exp OR 'liver disease'/exp) AND ('pharmacist'/exp OR 'clinical pharmacy'/exp OR 'hospital pharmacy'/exp)
CENTRAL	ID Search<br>#1 MeSH descriptor: [Liver Transplantation] explode all trees<br>#2 ("liver transplant" or "liver transplantation" or "hepatic transplantation"):ti,ab,kw (Word variations have been searched)<br>#3 MeSH descriptor: [Liver Cirrhosis, Biliary] explode all trees<br>#4 ("liver" AND "cirrhosis"):ti,ab,kw (Word variations have been searched)<br>#5 MeSH descriptor: [Pharmacists] explode all trees<br>#6 MeSH descriptor: [Professional-Patient Relations] explode all trees<br>#7 ("pharmacist"):ti,ab,kw OR (clinical pharmacist):ti,ab,kw OR (hospital pharmacist):ti,ab,kw OR ("pharmacare"):ti,ab,kw OR ("medication therapy management"):ti,ab,kw (Word variations have been searched)<br>#8 #1 OR #2 OR #3 OR #4<br>#9 #5 OR #6 OR #7<br>#10 #8 AND #9

Supplement Table S1. Search terms that were used for this systematic review, adapted to each database.

1	No author details and/or abstract available
2	Duplicate
3	Meeting/ Conference abstract/ Case report
4	outpatient setting
5	Does not include Liver transplant/ Cirrhotic patients
6	Includes pediatric patients not separated from adult patients
7	Does not include DRP
8	Does not include pharmacist intervention
9	Other, specify

Supplement Table S2. Nine exclusion criteria for abstract screening. DRP= Drug-related problem.
